# Clinical significance and prognostic value of receptor conversion after neoadjuvant chemotherapy in breast cancer patients

**DOI:** 10.3389/fsurg.2022.1037215

**Published:** 2023-01-06

**Authors:** Yang He, Jing Zhang, Hui Chen, Ying Zhou, Liping Hong, Yue Ma, Nannan Chen, Weipeng Zhao, Zhongsheng Tong

**Affiliations:** ^1^ Department of Breast Cancer, Tianjin Medical University Cancer Institute and Hospital, National Clinical Research Center for Cancer, Key Laboratory of Cancer Prevention and Therapy, Tianjin, China; ^2^ Department of Breast Cancer, Tianjin Cancer Hospital Airport Hospital, Tianjin, China; ^3^ Department of Integrative Oncology, Tianjin Cancer Hospital Airport Hospital, Tianjin, China; ^4^ Department of Oncology, Characteristic Medical Center of PAP, Tianjin, China; ^5^ Center for Precision Cancer Medicine and Translational Research, Tianjin Cancer Hospital Airport Hospital, Tianjin, China; ^6^ The First Department of Breast Cancer, Tianjin Medical University Cancer Institute and Hospital, Tianjin, China

**Keywords:** breast cancer, biomarkers conversion, neoadjuvant chemotherapy, prognosis, endocrine therapy

## Abstract

The hormone receptor (HR) status and human epidermal growth hormone receptor 2 (HER2) status of patients with breast cancer may change following neoadjuvant chemotherapy (NAC). We retrospectively analyzed the clinical data of 294 patients with stage II/III breast cancer to evaluate the clinical significance and prognostic value of receptor transformation after NAC in breast cancer patients. Pathological complete response after NAC was achieved in 10.7% of patients. HR, estrogen receptor (ER), progesterone receptor (PR), HER2, and Ki-67 conversion rates were 9.2%, 6.5%, 13.0%, 4.4%, and 33.7%, respectively. Patients with stable HR (*P* = 0.01) and HER2 (*P* = 0.048) expression had more favorable overall survival (OS). Low or reduced Ki-67 expression was associated with better disease-free survival (DFS) (*P* < 0.001) and OS (*P* < 0.01). Multivariate analysis showed that the number of lymph nodes after NAC, HR conversion, and radiotherapy were independent prognostic factors for overall survival. HR conversion implied a higher risk of death [hazard ratio, 2.56 (95% confidence interval: 1.19–5.51); *P* = 0.016]. Patients with HR conversion after NAC who received endocrine therapy had better DFS (*P* = 0.674) and OS (*P* = 0.363) than those who did not receive endocrine therapy, even if the HR changed from positive to negative. In conclusion, pathological testing should be performed before and after NAC, and even patients with HR conversion after NAC might benefit from endocrine therapy.

## Introduction

Neoadjuvant chemotherapy (NAC) is the standard treatment for locally advanced breast cancer. It not only reduces the clinical stage to make inoperable patients operable, but also significantly improves the prognosis of patients with human epidermal growth factor receptor 2 (HER2) positive, triple-negative breast cancer (1). However, there are inconsistencies in the expression of estrogen receptor, progesterone receptor, HER2, and Ki-67 before and after NAC. The same phenomenon was observed between primary and metastatic lesions in patients with advanced breast cancer. Changes in hormone receptor (HR) and HER2 status occur in 33% and 15% of patients, respectively (2). Therefore, the American Society of Clinical Oncology/College of American Pathologists recommends testing for breast cancer recurrence and metastasis. However, similar recommendations have not been made in relation to NAC, and few studies have investigated the association of biomarker alterations with long-term survival outcomes. In this study, we investigated the clinical significance and prognostic value of receptor conversion after NAC in patients with breast cancer.

## Materials and methods

### Study population

**Figure 1:**
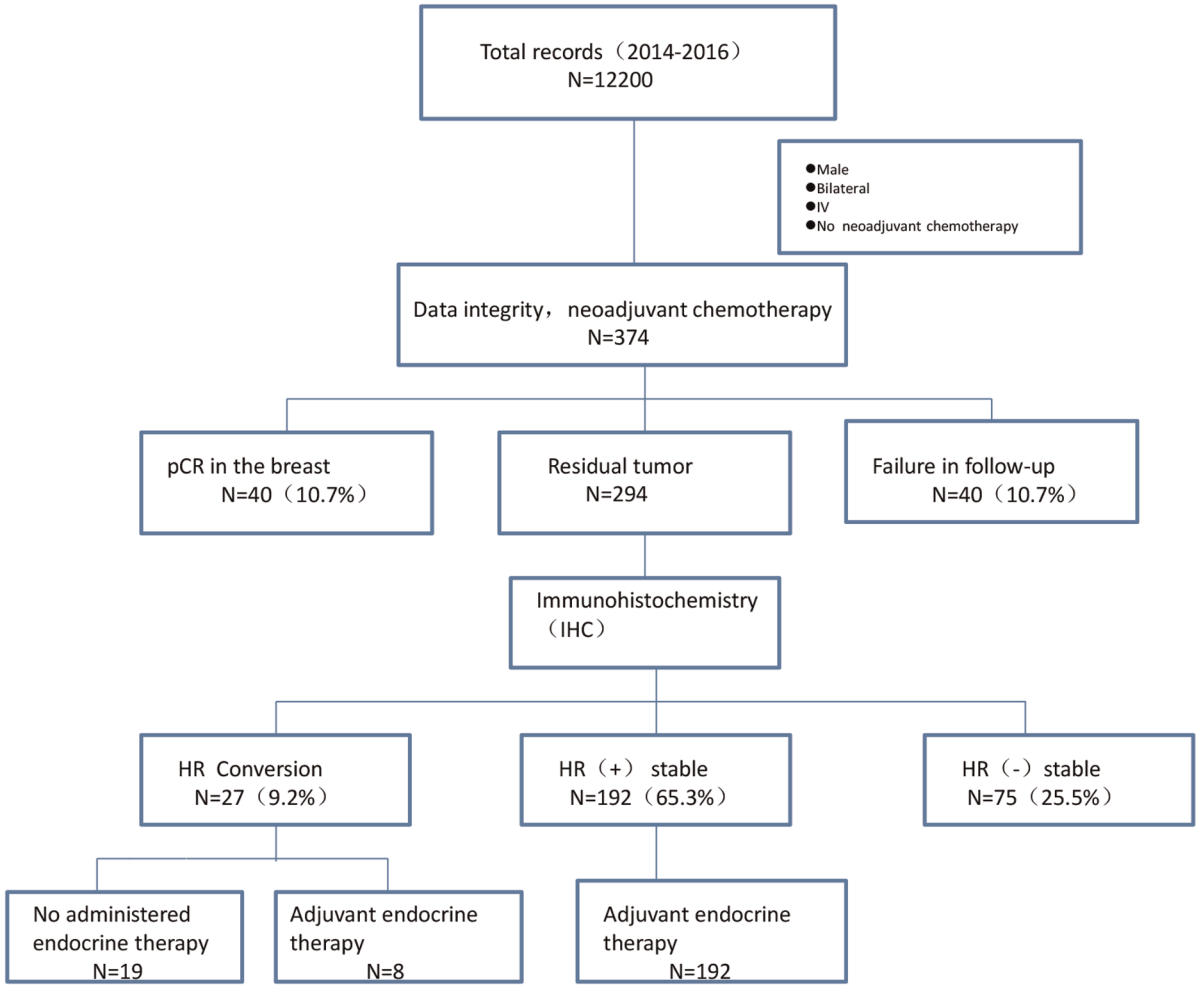
Flowchart of patient selection. Abbreviations: pCR, Pathological complete response; HR, hormone receptor.

Patients with breast cancer who received NAC at the Department of Breast Cancer, Tianjin Medical University Cancer Hospital, between January 2014 and December 2015 were enrolled. The main inclusion criteria were: (1) pathologically confirmed breast cancer after hollow-needle puncture before chemotherapy with known estrogen receptor, progesterone receptor, and HER2 status; (2) no treatment before NAC; and (3) available clinicopathological data. Patients were excluded if they (1) were pregnant or lactating, (2) had bilateral primary breast cancer, (3) were initially diagnosed with stage IV breast cancer, (4) had other primary malignancies, or (5) had severe organic heart or lung disease (Figure 1).

### Treatment

NAC regimens included 2‒8 cycles of cyclophosphamide, epirubicin, and 5-fluorouracil; epirubicin and cyclophosphamide; docetaxel and rubicin; docetaxel and carboplatin; docetaxel, epirubicin, and cyclophosphamide; or “other” regimens. After surgery, the treating physician determined whether to supplement the regimen with additional cycles of NAC, according to the patients' condition. After NAC, patients with indications for radiotherapy received radiotherapy. Endocrine therapy was performed for ≥5 years, depending on the outcome of discussions between patients and clinicians regarding their pathology.

### Immunohistochemistry

All pathological and immunohistochemical findings were independently evaluated by two experienced pathologists. Immunohistochemical staining sections were observed under the light microscope, and 10 visual fields were randomly selected under 10 × 40 high power microscope. Estrogen/progesterone receptor positivity was defined as ≥1% of positively stained tumor cells. HER2 status was determined according to the American Society of Clinical Oncology/College of American Pathologists 2013 guidelines. The tumor was considered HER2 positive if the primary tumor was scored as 3+ or 2+ by immunohistochemistry and confirmed by fluorescence *in situ* hybridization (FISH). Scores of 0 and 1+ were considered HER2 negative. A Ki-67 index ≥14% indicates high expression, whereas a Ki-67 index <14% indicates low expression. The following antibodies were used for immunohistochemistry: estrogen receptor (ready to use, clone # EP1, Dako), progesterone receptor (TA802606, clone OTI2E2, ZSGB-BIO), HER2 (ready to use, clone number 4B5, VENTANA), and Ki-67 (RMA-0542, SP6, Lab Vision Corporation).

### Clinical efficacy

Clinical efficacy was categorized as complete response, partial response, stable disease, or progressive disease, according to the Response Evaluation Criteria in Solid Tumors (version 1.1). Pathological complete response was defined as the absence of invasive cancer in the breast and axilla.

### Histopathological criteria for assessment of therapeutic response

The histopathological response to NAC was evaluated as follows: grade 0, no response; grade 1, slight response (marked changes in less than two-thirds of cancer cells); grade 2, marked response (marked changes in more than two-thirds of cancer cells with only a small number of invasive cancer cells remaining); or grade 3, complete response (necrosis and/or disappearance of all tumor cells and/or replacement of cancer cells by granulation and/or fibrosis) (3).

### Follow-up

Follow-up was performed by a combination of telephone and inpatient or outpatient visits until death or January 1, 2021. The primary outcomes were disease-free survival (DFS) and overall survival (OS). DFS was defined as the time from the date of diagnosis to the date of recurrence or the date of a second primary cancer diagnosis (including contralateral breast cancer). OS was defined as the time from the date of diagnosis to the date of death or last follow-up.

### Statistical analyses

Data were compared using the *χ*^2^ test or Fisher's exact test. Survival curves were estimated using the Kaplan‒Meier method and compared using the log-rank test. Univariate and multivariate analyses were performed using the Cox proportional hazards model (backward stepwise selection). All statistical analyses were conducted using SPSS 22.0 software. Statistical significance was set at *P *< 0.05. GraphPad Prism 7 was used for graphical representation.

## Results

### Clinical characteristics

Between January 1, 2014, and December 31, 2015, we selected 374 patients eligible for NAC from 12,200 breast cancer patients. The pathological complete response rate achieved with NAC after resection of breast cancer was 10.7% (40 of 374 patients). In addition, 40 patients who were lost to follow-up were excluded; thus, a total of 294 patients were enrolled. The clinical characteristics are summarized in Table 1. Of the 294 patients included in this study, 138 (46.9%) were <50 years of age and 166 (56.5%) were premenopausal. Two hundred and fifty-six patients (87.1%) had invasive ductal carcinoma, 14.3% had triple-negative breast cancer, >60% were HR positive, 16% were HER2 positive, and 90% received anthracycline combined with taxane-based chemotherapy. The median follow-up time was 72.3 (range, 7.1‒88.4) months. The median survival time was 84 months, with 3- and 5-year survival rates of 90.6% and 85.8%, respectively.

**Table 1:** Patient and tumor characteristics.

Characteristic	*N*	%
Age (years)		
<35	26	8.8
35–49	112	38.1
≥50	156	53.1
Menopausal status		
Premenopausal	166	56.5
Postmenopausal	128	43.5
Primary lesion site		
Left	147	50.0
Right	147	50.0
Family history		
Yes	79	26.9
No	215	73.1
Histological type		
Invasive ductal carcinoma	256	87.1
Other	38	12.9
Before NAC		
Tumor stage		
T1	21	7.1
T2	143	48.6
T3	74	25.2
T4	56	19.1
Node status		
Positive	259	88.1
Negative	35	11.9
Estrogen receptor status		
Positive	201	68.4
Negative	93	31.6
Progesterone receptor status		
Positive	174	59.2
Negative	120	40.8
HER2 status		
Positive	86	29.3
Negative	208	70.7
Ki-67 index		
High (≥14%)	270	91.9
Low (<14%)	24	8.1
Molecular typing		
Luminal A/B	205	69.7
HER2	47	16.0
Triple-negative breast cancer	42	14.3
After NAC		
Tumor grade		
I	4	1.4
II	86	29.2
III	7	2.4
N/A	197	67
Pathological therapeutic response		
1	125	42.5
2	92	31.3
3	21	7.1
N/A	56	19.1
Tumor stage		
T1	108	36.8
T2	138	46.9
T3	45	15.3
T4	3	1.0
Node metastasis		
0	63	21.4
1	102	34.7
2	76	25.9
3	53	18
Vascular invasion		
Yes	259	88.1
No	35	11.9
Estrogen receptor status		
Positive	196	66.7
Negative	98	33.3
Progesterone receptor status		
Positive	174	59.2
Negative	120	40.8
HER2 status		
Positive	78	26.5
Negative	216	73.5
Ki-67 index		
High (≥14%)	191	65.0
Low (<14%)	103	35.0
NAC regimen		
Taxane	10	3.4
Anthracycline	19	6.5
Anthracycline and taxane	265	90.1
NAC cycles		
1–2	28	9.5
3–4	98	33.3
5–6	131	44.6
>6	37	12.6
Adjuvant radiotherapy		
Yes	224	65.3
No	70	34.7
Adjuvant endocrine therapy		
Yes	192	65.3
No	102	34.7
Clinical response		
Partial response	176	59.9
Stable/progressive disease	118	40.1
Estrogen receptor conversion		
Negative to negative	86	29.3
Positive to positive	189	64.2
Positive to negative	12	4.1
Negative to positive	7	2.4
Progesterone receptor conversion		
Negative to negative	101	34.3
Positive to positive	155	52.7
Positive to negative	19	6.5
Negative to positive	19	6.5
HR conversion		
Negative to negative	75	25.5
Positive to positive	192	65.3
Positive to negative	13	4.4
Negative to positive	14	4.8
HER2 conversion		
Negative to negative	206	70.1
Positive to positive	75	25.5
Positive to negative	10	3.4
Negative to positive	3	1
Ki-67 index		
Low to low	14	4.8
High to high	181	61.5
High to low	89	30.3
Low to high	10	3.4

HER2, human epidermal growth factor receptor 2; HR, hormone receptor; N/A, not available; NAC, neoadjuvant chemotherapy.

### Receptor conversion

Immunohistochemistry detected 294 cases of residual tumor after NAC. HR status remained positive in 65.3% of patients and negative in 25.5%. In 13 (4.4%) of 294 patients, HR status changed from positive to negative after neoadjuvant therapy, and 4.8% (14/294) patients changed from negative to positive. The conversion rates of estrogen receptor, progesterone receptor, HER2, and Ki-67 status were 6.5%, 13.0%, 4.4%, and 33.7%, respectively (Table 1). Progesterone receptor was converted more readily than estrogen receptor. The loss of estrogen receptor and progesterone receptor in NAC was observed in 27.1% and 30.6% of patients, respectively, while the acquisition of estrogen receptor and progesterone receptor in NAC was observed in 23.1% and 21.1% of patients, respectively. These results show that the loss of HR in NAC was more common than acquisition.

After excluding menopausal status, tumor stage, lymph node status, vascular invasion, tumor histological grade, pathological treatment response, the number of lymph nodes after NAC, NAC regimen, the number of NAC cycles, and HER2 and Ki-67 status, changes in HR status occurred more frequently in patients <35 years of age or with a slight therapeutic response (grade 1) (Table 2). In addition, 13 patients received NAC combined with trastuzumab. In three patients, HER2 status changed from positive to negative (*P *= 0.019). The addition of anti-HER2 therapy was more likely to lead to loss of HER2 positivity.

**Table 2:** Comparison of the clinicopathological characteristics between the HR concordant and disconcordant groups.

Characteristic	HR concordant	HR disconcordant	*χ* ^2^	*P*-value
Age (years)			8.018	0.018*
<35	20 (7.5%)	6 (22.2%)		
35–49	106 (39.7%)	6 (22.2%)		
≥50	141 (52.8%)	15 (55.6%)		
Menopausal status			1.747	0.186
Premenopausal	154 (57.6%)	12 (44.4%)		
Postmenopausal	113 (42.4%)	15 (55.6%)		
Tumor stage			5.972	0.113
T1	21 (7.9%)	0 (0.0%)		
T2	130 (48.7%)	13 (48.1%)		
T3	63 (23.6%)	11 (40.8%)		
T4	53 (19.8%)	3 (11.1%)		
Node metastasis			3.715	0.294
0	33 (12.4%)	2 (7.4%)		
1	135 (50.6%)	18 (66.7%)		
2	77 (28.8%)	4 (14.8%)		
3	22 (8.2%)	3 (11.1%)		
Vascular invasion			0.573	0.449
Yes	33 (12.4%)	2 (7.4%)		
No	234 (87.6%)	25 (92.6%)		
Tumor grade			1.854	0.603
I	3 (1.1%)	1 (3.7%)		
II	77 (28.9%)	9 (33.3%)		
III	6 (2.2%)	1 (3.7%)		
N/A	181 (67.8%)	16 (59.3%)		
Pathological therapeutic response			6.008	0.05
1	109 (50.2%)	16 (76.2%)		
2	89 (41.1%)	3 (14.3%)		
3	19 (8.7%)	2 (9.5%)		
Post-NAC Tumor stage				
T1	99	9	0.583	0.846
T2	125	13		
T3	40	5		
T4	3	0		
Post-NAC node metastasis			1.314	0.726
0	58 (21.7%)	5 (18.5%)		
1	93 (34.8%)	9 (33.3%)		
2	70 (26.2%)	6 (22.2%)		
3	46 (17.3%)	7 (26.0%)		
NAC regimen			4.301	0.116
Anthracycline	15 (5.6%)	4 (14.8%)		
Taxane	10 (3.7%)	0 (0.0%)		
Anthracycline and taxane	242 (90.7%)	23 (85.2%)		
NAC cycles			1.211	0.750
1–2	24 (9.0%)	4 (14.8%)		
3–4	90 (33.7%)	8 (29.6%)		
5–6	120 (44.9%)	11 (40.8%)		
>6	33 (12.4%)	4 (14.8%)		
Ki-67 index			0.789	0.374
Low	23 (8.6%)	1 (3.7%)		
High	244 (91.4%)	26 (96.3%)		
HER2 status			1.946	0.163
Positive	76 (28.1%)	10 (41.7%)		
Negative	194 (71.9%)	14 (58.3%)		

HER2, human epidermal growth factor receptor 2; HR, hormone receptor; N/A, not available; NAC, neoadjuvant chemotherapy.

* *P *< 0.05.

### Prognostic effects of changes in HR, HER2, and ki-67 Status

**Figure 2:**
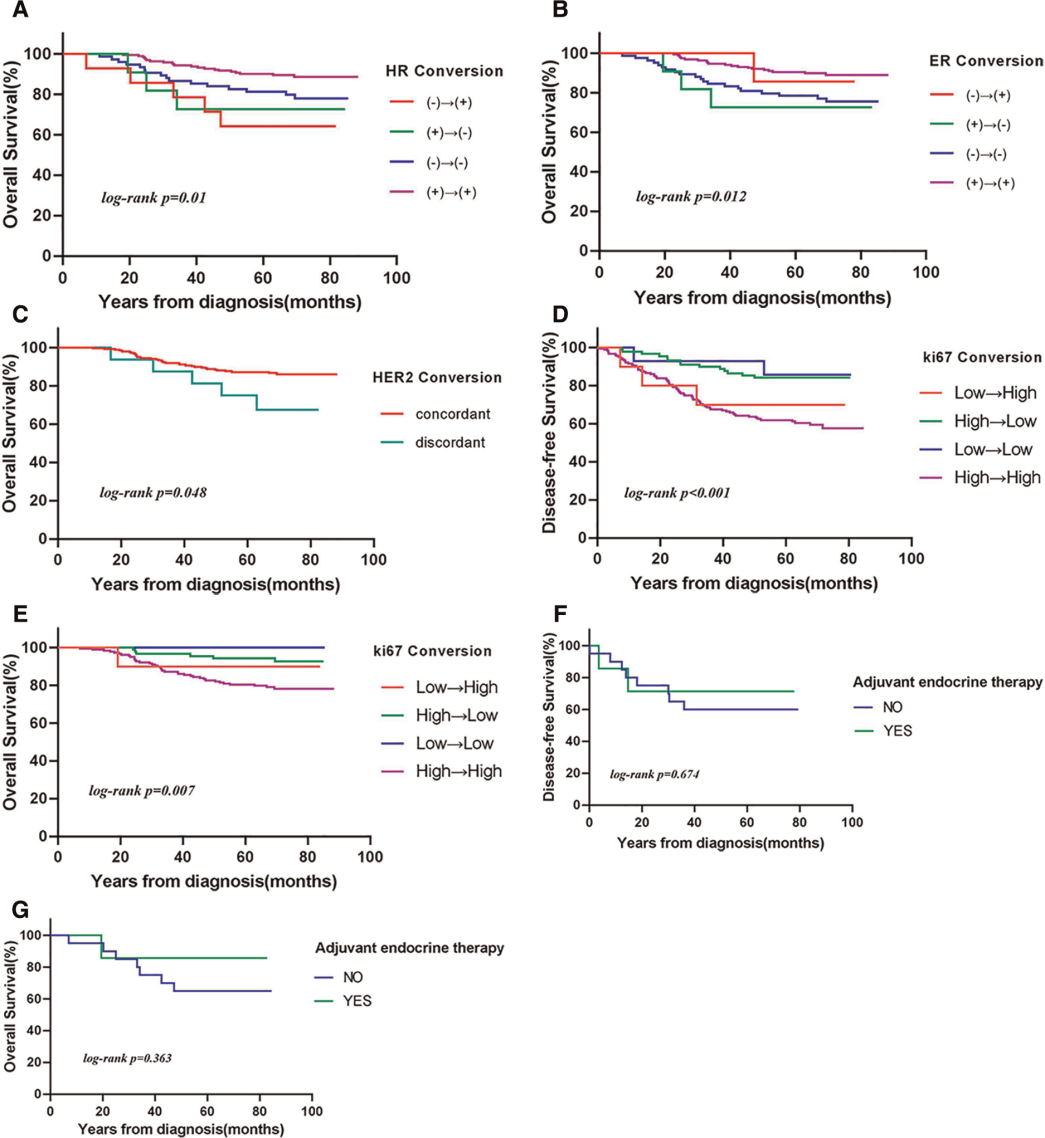
Kaplan–Meier curves of (**A–C,E,G**) overall survival (OS) and (**D,F**) disease-free survival (DFS) according to (**A**) hormone receptor (HR) conversion (*P *= 0.01), (**B**) estrogen receptor (ER) conversion (*P *= 0.012), (**C**) human epidermal growth factor receptor 2 (HER2) conversion (*P *= 0.048), (**D,E**) Ki-67 conversion [(**D**) DFS: *P *< 0.001; (**E**) OS: *P *= 0.007], and (**F,G**) HR conversion in patients treated with endocrine therapy [(**F**) DFS: *P *= 0.674; (**G**) OS: *P *= 0.363].

The 5-year OS rate was higher in patients with unchanged HR status than in those with changed HR status (86% vs. 70%, respectively). Patients with stable HR (*P *= 0.01) and HER2 (*P *= 0.048) expression had more favorable OS, but not DFS (Figure 2). Patients who remained estrogen receptor positive had significantly better OS than those who changed from estrogen receptor positive to estrogen receptor negative, and patients who remained estrogen receptor negative had significantly worse OS than those who changed from estrogen receptor negative to estrogen receptor positive (*P *= 0.012). Furthermore, the OS was longer in the ER status gain group than in the ER status loss group. Low or reduced Ki-67 expression after NAC was associated with better OS (*P *< 0.01) and DFS (*P *< 0.001). In patients with loss of Ki-67 expression, the 5-year OS estimates for patients whose tumors’ Ki-67 percent changes were ≥50% were significantly greater than those for patients whose tumors’ Ki-67 percent changes were <50% (*P = *0.01).

In univariate analysis, the number of lymph nodes after NAC, HR/HER2 conversion, Ki-67 status, radiotherapy, and endocrine therapy were significantly associated with survival (Table 3). In multivariate analysis, the number of lymph nodes after NAC [hazard ratio (95% confidence interval)] [2.58 (1.39‒4.75)] (*P *< 0.01), HR conversion [2.56 (1.19‒5.51)] (*P *= 0.016), and radiotherapy [0.25 (0.13‒0.50)] (*P *< 0.001) were independent prognostic factors for OS. Changes in HR status increased the risk of death 2.56-fold.

**Table 3:** Univariate and multivariate analysis of OS and DFS in patients receiving NAC.

Characteristic	Disease-free survival				Overall survival			
	Univariate analysis HR (95% CI)	*P*	Multivariate analysis HR (95% CI)	*P*	Univariate analysis HR (95% CI)	*P*	Multivariate analysis HR (95% CI)	*P*
Post-NAC node metastasis		<0.001*		<0.001*		0.003*		0.002*
N0‒1	1		1		1		1	
N2‒3	2.418 (1.584-3.690)		2.431 (1.540-3.839)		2.506 (1.361-4.615)		2.580 (1.399-4.757)	
HR status		NS		NS		0.025*		0.016*
Concordant					1		1	
Discordant					2.401 (1.118-5.157)		2.562 (1.191-5.513)	
HER2 status		NS		NS		0.048*		NS
Concordant					1			
Discordant					2.495 (0.977-6.370)			
Ki-67 status		0.001*		0.002*		0.008*		NS
Concordant	1		1		1			
Discordant	0.394 (0.233-0.668)		0.399 (0.226-0.705)		0.338 (0.151-0.756)			
Adjuvant radiotherapy		NS		0.034*		0.005*		<0.001*
No			1		1		1	
Yes			0.588 (0.360-0.959)		0.422 (0.233-0.767)		0.257 (0.130-0.507)	
Adjuvant endocrine therapy		NS		NS		0.001*		NS
No					1			
Yes					0.385 (0.214-0.694)			

CI, confidence interval; DFS, disease-free survival; HER2, human epidermal growth factor receptor 2; HR, hazard ratio; HR, hormone receptor; NAC, neoadjuvant chemotherapy; NS, not significant; OS, overall survival.

* *P *< 0.05.

### Survival analysis of receptor conversion treatment

Whether therapy management based on receptor conversion has an effect on survival has not yet been elucidated. We analyzed the effect of endocrine therapy on survival in patients with changes in HR status after NAC. Among 27 patients, eight received endocrine therapy. The 5-year OS and DFS rates were significantly higher in patients who received endocrine therapy than in those who did not (86% vs. 65% and 71% vs. 60%, respectively), although the differences were not statistically significant (Table 4). Similar results were obtained when HR positivity was defined as ≥10%.

**Table 4:** Relationship between changes in pathological markers and 3- and 5-year OS and DFS rates.

	*N*	DFS		OS	
		3-year	5-year	3-year	5-year
Overall	294	73%	68%	90%	85%
HR					
Concordant	267	73%	68%	90%	86%
Discordant	27	63%	63%	70%	70%
Estrogen receptor					
Concordant	275	73%	68%	88%	85%
Discordant	19	68%	68%	79%	79%
Progesterone receptor					
Concordant	256	72%	68%	89%	85%
Discordant	38	74%	64%	79%	78%
HER2					
Concordant	281	73%	68%	89%	86%
Discordant	13	69%	69%	81%	66%

DFS, disease-free survival; HER2, human epidermal growth factor receptor 2; HR, hormone receptor; OS, overall survival.

## Discussion

Several studies have examined changes in biomarkers after NAC. A meta-analysis (4) showed that NAC could significantly alter estrogen and/or progesterone receptor status, while HER2 status remained relatively stable. In our study, after NAC, estrogen receptor status changed from positive to negative in 4.1% of patients and from negative to positive in 2.4% of patients. Furthermore, progesterone receptor status changed from positive to negative in 6.5% of patients and from negative to positive in a further 6.5% of patients. HER2 status changed from positive to negative in 3.4% of patients and from negative to positive in just 1.0% of patients. These results are consistent with the findings of a large Japanese retrospective study (5). In the present study, progesterone receptor was more prone to conversion than estrogen receptor. We found that loss of HR in NAC was more common than acquisition. Further analysis showed that changes in HR status occurred significantly more frequently in patients <35 years of age or with a slight therapeutic response (grade 1).

The mechanism of the conversion of HR status after NAC is complex. Core-needle biopsy and excisional biopsy are generally considered highly consistent in detecting changes in estrogen and/or progesterone receptor status (6–8). The main reasons for the change in HR status include the small amounts of core-needle biopsy material, which may not accurately reflect the tumor microenvironment, as well as technical problems in the detection of immune components. In addition to false positives and false negatives caused by the detection method, there may be mechanistic changes in HR status after NAC. Tumor cells differ in their sensitivity to chemotherapy. HR negative cells are more sensitive to chemotherapy, whereas HR positive cells are preserved (9). Chemotherapy inhibits ovarian function in premenopausal women, reduces the levels of circulating hormones, and downregulates estrogen and/or progesterone receptor, leading to hormone-independent growth (10). This explains the change in HR status from positive to negative after NAC. HR conversion from negative to positive may be explained by the fact that the cells were originally derived from well-differentiated HR positive breast cancer cells and had returned to their original state during chemotherapy or targeted therapy. Nie et al. (11) transformed triple-negative breast cancer into luminal breast cancer using CDK2/EZH2 inhibitors and reactivated estrogen receptor alpha expression so that endocrine therapy could be performed. Another study (12) showed that chemotherapy upregulated protein expression in the nuclei of tumor cells, leading to enhanced expression or re-expression of HRs and changes in HR status.

Kaplan‒Meier analysis showed that stable HR expression was associated with significantly better OS. Patients aged <35 years or with a slight therapeutic response (grade 1) were more likely to undergo HR conversion. These two indicators represent a poor prognosis and may explain the higher survival rate of patients with unchanged HR status. In a meta-analysis, Li et al. (13) showed that compared with patients who remained HR positive, those who became HR negative had worse OS and DFS. Moreover, patients whose HR status changed from negative to positive had better OS and DFS than those who remained HR negative. These findings are inconsistent with those of the current study, possibly due to differences in the cutoff values used. Ahn et al. (14) reported that negative conversion of estrogen receptor and progesterone receptor status after primary systemic therapy was associated with reduced DFS. In a prospective database study (15), any change in HR status resulted in a worse prognosis compared with stable HR status. This was consistent with another study (16) and supported our conclusion. After excluding confounding factors, such as endocrine therapy and HER2 and Ki-67 status, multivariate analysis identified the number of lymph nodes after NAC, HR conversion, and radiotherapy as independent prognostic factors for survival. Changes in HR status significantly increased the risk of death [hazard ratio, 2.56 (95% confidence interval: 1.19‒5.51); *P *= 0.016].

It is unclear whether patients with altered HR status after NAC for breast cancer could benefit from endocrine therapy. Wu et al.) (17) showed that although HR status changed from positive to negative after NAC, patients still benefited from endocrine therapy. However, the benefit was not significant. Hirata et al. (18) reported that in patients whose HR status changed after NAC, the endocrine therapy group had better OS and DFS than the no endocrine therapy group, and that changes in HR status did not affect long-term survival. In this study, HR status changed after NAC in 27 patients, eight of whom received endocrine therapy. The 5-year OS and DFS rates were higher in patients who received endocrine therapy than in those who did not (86% vs. 65% and 71% vs. 60%, respectively), possibly due to the small sample size, although the differences were not statistically significant. We still suggest that endocrine therapy should be considered for patients with only one positive HR status before and after NAC.

Amplification and/or overexpression of HER2 occurs in 14%–30% of all breast cancer cases (19), HER2 status is mainly evaluated by immunohistochemistry and FISH. Although the results obtained by immunohistochemistry and FISH are highly concordant, FISH is more reproducible and stable than immunohistochemistry (20). In our study, due to medical insurance policy issues, only a small number of patients used anti-HER2 targeted therapy. In particular, 13 patients received NAC combined with trastuzumab. The HER2 status of three patients changed from positive to negative. The addition of anti-HER2 therapy was more likely to lead to loss of HER2 positivity. Ignatov et al. (21) showed that the differences in HER2 status before and after NAC only related to anti-HER2 therapy. Moreover, the addition of pertuzumab to trastuzumab increased the rate of HER2 loss from 47.3% to 63.2%. A retrospective study of a prospective database (22) showed that HER2 loss was more common in the paclitaxel, carboplatin, and trastuzumab group than in the paclitaxel and carboplatin group. In a study of patients with HER2 positive gastric cancer (23), approximately one-third of patients lost HER2 expression after trastuzumab-based treatment. These findings support a positive association between increased loss of HER2 positivity and anti-HER2 therapy.

The use of anti-HER2 therapy in NAC not only increases the pathological complete response rate in HER2 positive patients but also improves survival. In an analysis of a prospective database (24), patients with loss of HER2 positivity had a higher risk of recurrence than those with stable HER2 expression. Mittendorf et al. (25) showed that patients with stable HER2 expression had better recurrence-free survival. The results of these studies are not consistent, and we support the latter conclusion more; patients with stable HER2 expression had a significantly lower risk of death than those with altered HER2 expression (*P *= 0.048).

Ki-67 is used to assess proliferation (26). Studies (27, 28) have shown that Ki-67 expression is an independent prognostic factor for OS in patients with breast cancer. High Ki-67 expression is associated with an increased risk of death. Ki-67 expression is significantly reduced after NAC, and patients with high Ki-67 expression in residual tumors have poor OS and DFS. In this study, low or reduced expression of Ki-67 after NAC was associated with better OS (*P *= 0.007) and DFS (*P *< 0.001). Our results show that reduced Ki-67 expression predicts a better prognosis after NAC in patients with breast cancer.

Inevitably, our study has some limitations. First, it was a retrospective, non-randomized database study in which there were some uncontrollable factors, such as chemotherapy regimen and choice of anti-HER2 therapy. Second, our data were obtained from a hospital database, with no centralized reassessment of estrogen receptor, progesterone receptor, or HER2 status. Moreover, the incidence of receptor switching is infrequent, and the prognostic value of receptor switching and treatment selection is best assessed in prospective trials.

## Conclusions

Our study has important clinical implications, although it is retrospective. In this study, changes in HR status induced by NAC can be used as a prognostic factor for DFS and OS; stable HR and HER2 status were associated with better OS. We strongly recommend that pathological testing be performed before and after NAC. More importantly, patients with altered HR status after NAC might benefit from endocrine therapy, even if the HR changes from positive to negative.

## Data Availability

The original contributions presented in the study are included in the article/Supplementary Material, further inquiries can be directed to the corresponding author/s.
